# Arsenic in Beer

**Published:** 1901-05-04

**Authors:** A. H. Bampton


### Hospital Clinics and Medical Progress.

### ARSENIC IN BEER.

"A History of an Epidemic and its Lessons." Paper read before the Yorkshire Branch of the British Medical Association at York by A. H. Bampton, M.D.

Some time in June last, in Ilkley-in-Wharfedale, it was noticed that an unusual number of patients complained of numbness, prickings, formication, and burning pains in hands and feet, and our attention was attracted, on our daily professional rounds, to a number of folk with inflamed eyes who limped painfully along the roads. Many of these sufferers belonged to the working class, and did not consider themselves sufficiently ill to seek medical advice. Those of them who came under my own immediate notice were either Post Office or railway employés, or worked at the local brewery.

At the onset of the epidemic the opinion was hazarded that these cases of peripheral neuritis must be the after-effects of unnoticed and slight attacks of diphtheria. Personally, I confess, that at first I laid to the charge of influenza all these symptoms, the more particularly because of the marked catarrhal head symptoms, persistent pharyngitis, associated with marked weakness of the lower extremities exhibited—a group of symptoms sufficiently like those seen in catarrhal influenza to deceive the very elect. In addition to which I had under observation at the same time an unusual number of cases of neuritis, in ladies, affecting one limb only, mostly the right arm, all of which had undoubtedly followed attacks of genuine influenza. But, by-and-by, we had quite a number of acute skin lesions to deal with. A man's foot would suddenly become red, hot, and painful, and large bullæ would appear on it (Erythromelalagia). "Herpes" in various parts of the body, and urticarial rashes in July and August were seen. The public attributed the sore feet to excessive sweating during the humid atmosphere that prevailed in July and August, and the patients resented the medical opinion that these skin affections were certainly of dietetic origin.

And as some of the cases of peripheral neuritis were thus attacked, to whom the alcoholic habit was attributed, and we further found that beer-drinking was a factor common to all these cases—groping rather blindly at the truth—we advised patients to stop beer-drinking as a measure of precaution; and to such effect, that very little ale was drunk during the latter months of the year, and the local brewery, in consequence, almost ceased brewing six weeks before arsenic was found to be the *fons et origo mali.*

I was at the time making inquiries as to whether some "preservative" that would act as an irritant was| being used, such as "formalin," and was told by one of the directors who consulted me that a thorough analysis of all the ales was to be made immediately. "Formalin" was the only preservative of which I knew that could possibly produce irritation in the eyes, skin, and nerves. And I was not sure of this, and suggested that an exhaustive analysis should be made for any other irritant. The brewery authorities were quite open in the matter, and courted every inquiry, honestly believing nothing was the matter with their brewings. They said, "We use nothing but hops, malt, and brewers' sugar, and those of the best." Thus the matter rested, waiting for the analyst's report.

Fortunately public opinion took our advice and condemned the beer by this time, and drank very little of it. And to this empirical judgment is due the fact that so few of our cases became serious or dangerous to life.

It was well on in November when my attention was called to Dr. Reynolds' discovery of arsenic in Manchester beer. Search was then immediately made for arsenic in our hops, and finding none, the sugar was analysed, and arsenic to the extent of gr. iij to the lb. was found in it, and subsequently in most of the ales in varying proportions. The analyses were conducted on behalf of the brewery by Messrs. Rimmington, of Bradford, whose results were invariable confirmed by the *Lancet's* analyst and the chemist of the Clinical Investigation Society, to whom I also sent samples. The irony of the situation at this juncture was that the long-expected report from the brewer's analyst arrived to say "that all the ales were remarkably pure"! This report was followed by a message that, hearing of the arsenic scare, the analysts would now look for As. Then came a report that none was found. On their being told that other analyses did not agree with theirs, they made another search and found it, and excused themselves by saying that an assistant had conducted the former experiments.

It appears that brewers' analysts look only for what ought to be in beer, and do not look for extraneous matters as a toxicologist would most surely do. We had, therefore, waited in vain for any guidance from an expert, and it shows how limited is the value of many public analysts' reports. That leads me to remark how insecure is our position with regard to the purity of our foods and drugs. It is notorious that up to quite recently there was not a phosphate of soda on the market that did not contain As—phosphate of soda which we administer to infants and delicate patients as a *mild* and safe aperient!—and I am told that there is but little citric acid sold that does not contain a trace of lead! "Sugar of milk" has been, I know, adulterated with "alum," and been the principal cause of obstinate constipation in bottle-fed infants.

"Boric acid and iodoform," unless used in their crystalline form, may be loaded with foreign matter and be the cause of sepsis in wounds. But to return to our beer. I will briefly relate three or four typical cases that represent groups; and the way the As affected them.

Type 1.—A. L., employed at the brewery, states that in the summer, "after taking a glass of beer with his dinner, he had pain in his stomach, vomited, and was purged. This occurred again at supper, with which meal he also took beer. He then had some tingling of hands and feet, followed by desquamation, some herpes over abdomen, and intolerable skin irritation at night. Leaving off beer, all these symptoms disappeared, but he broke out in acute general eczema, which yielded to local treatment. I may remark here that double the usual quantity of "glucose" was being used at this time in the brewing, and hence the acute and immediate symptoms manifested. It is possible that $\frac{1}{6}$ gr. As was then in a glass of beer, certainly not less than the equivalent of liq. As ♏ vi. (Fowler's sol.).

Type 2.—C. V. came under notice for pharyngitis, conjunctivitis, tingling, numbness, and loss of power in hands, feet, and legs, with dropped big toe and loss of knee jerks. This patient drank beer, but had not the appearance nor the reputation of being a big drinker. C. V. recovered from one attack under iodide of potassium and rest in bed, but relapsed as soon as he resumed work and beer.

The second attack was more persistent, but a change of air hastened his recovery. There remains to this day still some dropping of the left big toe. This patient's hands looked hornified (keratosis) and his feet swollen, red, and tender. He suffered from obstinate constipation, and this constipation was a puzzling feature in many other cases, almost negativing idea of As. It may be that the sugar in the beer combines with the As to form some definite compound, as it is known to do with calcium and lead, which compound may have a secondary constipating effect. Or the constipation may be a feature of the neuritis, due to partial paresis of the intestinal nerves, as is known to occur in the peripheral neuritis due to excessive use of sulphonal, in which condition obstinate constipation is also a prominent symptom.

Type 3.—M. R. is seized with violent pain in the left hypochondrium of a persistent character, associated with continued vomiting. The tongue is coated with white fur. The bowels at first purged, but subsequently constipated. There is great cardiac weakness and tendency to syncope. The skin has been noticed to have grown darker in colour of late. After a similar attack in the summer for which no cause could be assigned, the patient had an irritable skin eruption which left brown stains. This patient was very ill, and only improved when the catamenia was established. A relapse was brought on during the first attack after taking a glass of beer. There was no uterine flexion, nor lameness of any kind. I regard this case as an instance of the cumulative effect of a drug taking on violent action at the menstrual period, when drugs are ill borne. This patient drank three glasses of beer daily. They kept a cask of ale in the house. I had a specimen examined, and the analyst reported that there was a very appreciable quantity of As present, and this was confirmed by Messrs. Rimmington. This patient's husband was an interesting case whom Dr. Churton kindly saw with me.

Twelve years ago his testes were removed for tubercle. He had been able to do odd jobs ever since—gardening and the like. Although not robust, was not laid up until this summer, when he was employed as greenkeeper to one of the bowling greens. He became very weak in the legs. In a sunless summer he was noticeably tanned; his feet became blistered and desquamated, and his eyes red and weak. He could not wear his boots because his feet were so red and swollen and tender. At this time he was drinking five or six glasses of beer at the least most days.

He did not come under my care or give up work until the beginning of December, when he had synovitis of the left knee-joint; the effusion was purely serous. Patient was then bronzed all over, the pigment being most pronounced over the knees, ankles, about the genitals, pubes, and axillæ. There was no staining of the buccal mucous membranes, knee-jerks were absent, and there was great muscular tenderness.

There was some purulent expectoration, but no bacillus tuberculosis could ever be found in expectoration nor in his urine. Arsenic was looked for in his urine, but not found. Temperature at the last ranged about 100°. No physical signs of tubercle in chest. There was no mental hebetude, and great tendency to syncope and some retching. The patient died after a month's illness. Unfortunately no post-mortem could be obtained, and the coroner did not consider an inquest necessary. I certified, query tuberculosis of suprarenal capsules and arsenical poisoning from beer-drinking. I much regretted not being able to verify the diagnosis.

There are so many inquests to be held at Manchester and elsewhere in connection with arsenical poisoning, from which, I believe, we shall glean very valuable information which may modify our views, not only on alcoholic neuritis, but on bronzing of the skin. From evidence that we have received, sulphuric acid has been made from arsenical pyrites for many years past, and it is probable that arsenical contamination has to a less extent been present in some brewer's sugar from time to time, whenever the exigencies of the manufacturer prompted, or the custom of the trade permitted, arsenicated sulphuric acid to be supplied. The malt and hops have also been shown not to be above suspicion.

It is a curious circumstance of some significance that the London School of Neurologists have contended that alcoholic neuritis was the manifestation of spirit-drinking, and was not often seen in beer-drinkers, whilst the Manchester School held that peripheral neuritis was commonly and mostly seen in beer-drinkers. Some of the cases of neuritis were probably examples of the compound toxic effect of alcohol and As and influenza in varying proportions. We are getting to recognise more clearly that the human body may have more than one specific germ or toxin at work or at rest, exercising their lethal effect at one and the same time. Similarly, to what we see takes place in our gardens, in the soil of which many varieties of both flowers and weeds can grow side by side or follow in successive crops. Again, one toxin may augment the effect of another toxin, just as drugs with similar physiological actions augment each the others action.

And just as when a patient is under the influence of lead a very slight indiscretion in diet is sufficient to produce an attack of gout, so when there is As in the beer small doses of the augmented fluid will produce peripheral neuritis, which singly neither the alcohol nor the As would be sufficient to produce. We must from this experience be more ready to recognise that uncomplicated, simple, pure, and classical varieties of disease are the exception rather than the rule, and are mostly met with in our text-books.

Not many women came under medical observation for various reasons—one was that they did not want to be known to be beer-drinkers, another reason was that they did not belong to sick clubs and would therefore have to pay for medical advice.

"Arsenic" is frequently given as a blood tonic, but this epidemic must shake our faith in its virtues, because, although so widely drunk, there have been no indications of improved blood value in any of the beer-drinkers, such as increased robustness or improvement in wind, in limb, which both they and their neighbours would have noticed, had the arsenicated beer had such a tonic effect upon them. In conclusion, I would remark that the public owe a deep debt of gratitude to Dr. Reynolds, which ought to be officially recognised.

